# First application of three‐dimensional light sheet fluorescence microscopy to human testicular tumors: New perspectives in histopathology

**DOI:** 10.1111/andr.70009

**Published:** 2025-02-12

**Authors:** Diana Pinkert‐Leetsch, Ahmad Kareem, Simon F. Merz, Marc Teckentrup, Alexander Fichtner, Felix Bremmer, Jeannine Missbach‐Guentner

**Affiliations:** ^1^ Department of Clinical and Interventional Radiology University Medical Center Göttingen Göttingen Germany; ^2^ Miltenyi Biotec, B.V. & Co. KG Germany Bielefeld Bergisch Gladbach Germany; ^3^ Department of Pathology University Medical Center Göttingen Göttingen Germany

**Keywords:** 3D virtual histology, autofluorescence, germ cell tumors, light sheet fluorescence microscopy, male reproductive organ, testis

## Abstract

**Background:**

Testicular tumors are among the most frequently diagnosed cancers in young men. The consequences of this diagnosis are orchiectomies, severely restricting fertility. For these young patients, a comprehensive diagnostics would be desirable, achieving a refined diagnosis and improved therapeutic patient stratification.

**Objective:**

The aim of this study was to use three‐dimensional (3D) light sheet fluorescence microscopy (LSFM) to analyze a complete testicular tumor punch at subcellular resolution—allowing a detailed diagnostic assessment of the entire punch.

**Materials and methods:**

Tissue punches (3  and 5 mm diameter) were taken from paraffin blocks of four miscellaneous testicular tumors. After deparaffinization and clearing using benzoic acid/benzyl benzoate, a label‐free LSFM autofluorescence imaging was performed. In addition, TO‐PRO‐3 nuclear stain was applied to several punches. After the scan, the samples were embedded in paraffin again and physically sectioned for conventional planar histology.

**Results:**

Based on the specific autofluorescence, not only the general morphology of the tumor tissue was identified in LSFM datasets, but also diagnostic features like infiltrations, papillary and pagetoid tumor cell formations, germ cell neoplasia in situ and azoospermia. Subcellular characteristics such as vacuolated cytoplasm and pleomorphic nuclei could be detected at maximum magnification. After nuclear staining, virtual H&E sections were reconstructed from the LSFM data and tomographically visualized across the entire punch. Subsequent histology and immunohistochemistry after LSFM analyses is possible.

**Discussion:**

LSFM analysis of testicular tumors enables the detailed 2D/3D analysis of an entire tumor punch for assessment of relevant tumor characteristics due to its intrinsic fluorescence or with specific nuclear staining.

**Conclusion:**

LSFM provides the technical basis for the analyses of complete testicular tumor biopsies, thus maximizing the spatial morphological and anatomical information. The subcellular 3D imaging of the tumor has the potential to identify new cancer imaging biomarkers that have additional diagnostic and prognostic value for patients.

## 1 INTRODUCTION

Germ cell tumors (GCTs) are among the most frequently diagnosed cancers in young men between the ages of 15 and 40 years.^1^, ^2^ GCTs account for 95% of all testis tumors and arise from a precursor lesion called germ cell neoplasia in situ (GCNIS), which is considered to originate from developmentally arrested primordial germ cells.^3^, ^4^ GCTs are divided into seminomas and non‐seminomas.^5^, ^6^ The group of non‐seminomas includes embryonal carcinomas, yolk sac tumors, teratomas, and choriocarcinomas.^4^ Non‐GCT tumors encompass tumors of the gonadal stroma, including Leydig cell tumors (LCT), Sertoli cell tumors, and other rare tumors. LCT are the largest subclass of these tumors and account for about 3%–5% of all adult testicular tumors.^7^ In addition to painless testicular enlargement, LCT also causes symptoms that can be attributed to an imbalance in the sex hormone production: gynecomastia, infertility, or subinfertility.^8^

The diagnosis of GCT and non‐GCT is made after a clinical examination, subsequent ultrasound imaging, curative surgery, and following histological analysis.^9^, ^10^, ^11^ The increased use of testicular ultrasonography (US) to assess these conditions has led to an increased number of incidentally detected small, non‐palpable lesions.^11^, ^12^

In this context, LCT, although rare in principle, account for approximately 45% of all incidental findings^12^, ^13^, ^14^ and are now part of a significant increase in the number of small non‐palpable lesions of the testis, particularly in infertile patients. The far‐reaching consequences of the diagnosis of a testicular tumor are often burdening orchiectomies, although the original wish of the patients was to restore their fertility.^11^, ^12^ In the course of the tumor therapy however, prior sperm cryopreservation is indicated for most patients^15^ because a transinguinal orchiectomy is the state‐of‐the‐art treatment to reduce the spread of tumor cells in the case of the testicular GCT. The histopathological examination for differential diagnosis and determination of the malignant potential of the tumor after an orchiectomy is the only way to make a correct subtyping of the tumor entity.^16^ For these patients in particular a comprehensive differential diagnosis would be desirable to enable the best‐tailored therapy, a precise assessment of the malignant potential of the tumor and therefore an accurate individual prognosis.

Tissue imaging using conventional histological methods provides useful information for characterizing tissue composition in routine clinical practice, but is limited to two‐dimensional (2D) imaging. Three‐dimensional (3D) imaging offers an improvement in capturing the spatial context of biological samples and can be realized by using the light sheet fluorescence microscope (LSFM). LSFM is a fluorescence microscopy technique to acquire optical sections within intact specimens. In LSFM, each plane is illuminated tomographically with a light sheet perpendicular to the detection, which significantly reduces the bleaching of the sample.^17^ In addition, LSFM is a microscopic method that is able to measure three‐dimensional volumes of biological samples.^18^ In contrast to confocal microscopy as another applicable technology for 3D imaging, LSFM excels in the size and speed of the 3D imaging, thus making it the far superior approach to image intact samples. Prior to LSFM, biological samples have to be prepared to match the refractive index to the medium in which the microscopic scans are performed, a process called tissue clearing.

Typically, fresh samples are formalin fixed and subsequently undergo either water‐based or solvent‐based tissue clearing. Here, we used benzyl alcohol/benzyl benzoate (BABB) to clear the tissue and ethyl cinnamate (ECi) for RI matching.^19^, ^20^, ^21^ One strategy to reliably increase the transparency of biological samples is solvent‐based clearing with BABB, which enables clearing of samples with a lot of extracellular connective tissue, such as kidney, pancreas, lung.^22^ BABB has also been successfully used to image the fresh tissue of murine testes, which were analyzed with LSFM for their autofluorescence properties during development.^23^ However, an emerging alternative approach is the use of already fixed and paraffinized tissue, which must first be deparaffinized before clearing. Studies on paraffin‐embedded material have been conducted, for example, for 3D LSFM analysis of the prostate and prostate cancer to explore the feasibility and analytical benefits.^24^ It is advantageous that entire tissue specimens can be scanned in retrospective studies and thus planar histologies of these specimens can be supplemented with spatial information. Tanaka and colleagues succeeded in detecting comprehensive patterns of intratumoral tumor heterogeneity in LSFM data of various human paraffin‐embedded tumor samples such as bladder, pancreas, head & neck and others, which were subsequently validated by immunohistochemistry.^25^

The aim of the present study was to clarify whether a 3D histological analysis of an entire tumor punch is possible after preparation and clearing of already paraffin‐embedded testicular tumors in LSFM, ideally providing complementary information to conventional planar histopathology and thus making an important contribution to comprehensive histopathological characterization.

Our data show: A) an adapted workflow of sample preparation from paraffin block to LSFM image. B) Descriptive, morphological analyses of testicular tumors of different genesis based on their autofluorescence. C) The additional use of nuclear staining for targeted visualization of different intratumoral cell populations, and D) The validation of the obtained LSFM data by subsequent histology and immunohistochemistry of the reparaffin‐embedded tumor samples.

## 2 MATERIAL AND METHODS

### 2.1 Sample origin

Paraffin blocks from Murine testis were taken from C57BL6/J mice; age 1.5 ‐ 4.5 month. The murine testis has been treated and paraffinized as previously described.^23^ Patient testicular tumor specimens were obtained as paraffin blocks from the Department of Pathology, University Medical Center (Goettingen, Germany). Pathological findings were made as part of each patient's diagnostic process and were summarized in Table 1.

**TABLE 1:** Clinical characteristics and findings of the used patient's specimen.

Patient number	Age at diagnosis	Diagnosis	Origin	Staging
1	31 years	Seminoma	Orchiectomy	pT1 NX L0 V0 Pn0 R0
2	33 years	Seminoma	Orchiectomy	pT2 NX L0 V1 Pn0 R0
3	27 years	Mixed tumor: Embryonal carcinoma (88%) Teratoma (10%) Yolk sac tumor (1%) Chorion carcinoma (1%)	Orchiectomy	pT2 NX L0 V1 Pn0 R0
4	45 years	Leydig cell tumor	Orchiectomy	pT1 NX L0 V0 Pn0 R0

### 2.2 Ethics statement

All animal experimental procedures were performed in compliance with the European (2010/63/EU) and German regulations on Animal Welfare and were approved by the administration of Lower Saxony (LAVES) {Nr. 33.9‐42502‐04‐17.2742}. The human material was used with the permission of the local ethics committee of the University Medical Center Göttingen (Ethics Vote 20/09/20 to FB) and in accordance with the Declaration of Helsinki.

### 2.3 Sample preparation

After initial histological evaluation of Hematoxylin/Eosin (H&E) stained 2 µm tissue sections of paraffin‐embedded testicular tumor tissue samples, areas of particular interest were selected. Thereafter, 3 mm, respectively 5 mm diameter punch biopsies were taken from the paraffin block and prepared for further analysis. The mice testes were taken as a whole.

The mice testes and some of the testicular tumor punch biopsies were deparaffinized and rehydrated in a descending alcohol series in order to embed them into gelan gum (Phytagel, MERCK KGaA; 0,7% in H_2_O) for better stabilization of the sample. The Phytagel‐embedded testes samples were then dehydrated in an ascending alcohol series and placed in BABB (ratio 1:3) to adjust the refractive index to prevent light scattering and light absorption in the immersion chamber of the microscope.^23^ Some of the tissue punches from the testicular tumors were additionally stained with the nuclear dye TO‐PRO‐3 (TO‐PRO™‐3 Iodide, ThermoFisher Scientific) after deparaffinization and descending alcohol steps (xylene, ethanol 100 %, ethanol 70 %), as described previously.^26^ The protocol was adapted by using a TO‐PRO‐3 concentration of 1:1000 and not counterstaining with eosin. Embedding in Phytagel was not carried out for these samples. Subsequently, the samples were also cleared with BABB at room temperature until transparency was achieved. Depending on the tissue composition, this was a period of 48 h to approximately 5 d for punch biopsies and up to 12 d for the entire murine testes. After analysis by LSFM in ECi, the samples were transferred back to BABB for storage until histological validation.

### 2.4 Light sheet fluorescence microscopy and data analysis

The cleared murine testes and testicular tumor punch biopsies were analyzed using the UltraMicroscope Blaze (Miltenyi Biotec B.V.& Co KG, Germany). The specimens were attached either by a screw or with superglue to the sample holder of the microscope. In order to fix the nuclear stained 3 mm tissue punches properly on to the holder, they were placed in pre‐cut and pre‐cleared Phytagel cubes. For the LSFM scanning procedure they were placed into the ECi filled cuvette, which was used as a non‐corrosive and non‐toxic alternative to BABB with comparable refractive index. Fluorescence imaging was done using an NKT SuperK Extreme white light laser of 0.6 W visible power (NKT Photonics A/S, Denmark) as a light source. The corresponding filter sets for excitation and emission used are listed in Table 2. The filter sets used for LSFM microscopy are listed in Section 3 according to their numbering in the table. The standard measurement parameters include a numerical aperture of 0.163 and a light sheet thickness of 3.9 µm. Depending on the objective (1.1×, 4×, 12×) used, the sample step size in the *Z*‐direction was either 2 µm or 4 µm, and the maximum resolution was between 4.8 and 0.5 µm. Images were captured using the ImSpector (version 7.5.2) software. The Blaze is equipped with a 4.2‐megapixel sCMOS camera with a 2.048 × 2.048 pixel resolution (PCO AG, Germany). LSFM data analysis was performed using Zeiss arivis Pro software (Carl Zeiss Microscopy Software, Germany).

**TABLE 2:** List of all filter combinations used for LSFM data acquisition.

Filter set	1	2	3	4	5
Excitation wavelength/ bandwidth in nm	470/40	520/40	595/20	630/30	710/75
Emission wavelength/ bandwidth in nm	525/50	585/40	650/50	680/30	810/90

Abbreviation: LSFM, light sheet fluorescence microscopy.

Image post‐processing was performed using a virtual Hematoxylin and Eosin (H&E) algorithm implemented with Halide in C++. For each image in the nuclei or extracellular channel, several refinement steps were conducted, including background subtraction, edge detection for image sharpening, and normalization. The normalization process involves adjusting an acquisition‐specific parameter to optimize saturation. Finally, each image channel is processed according to the following equation:

$$O\;\left(x,y\right)=e^{-c_{h,\;\mathrm{rgb}}\cdot H\left(x,\;y\right)}\;\cdot e^{-c_{e,\;\mathrm{rgb}}\cdot E\left(x,\;y\right)},$$

where H(x,y) and E(x,y) are the input images in RGB space (instead of Eosin, tissue autofluorescence in the green channel was taken) and O(x,y) is the output image, while $c_{h,\;\mathrm{rgb}}$ and  $c_{e,\;\mathrm{rgb}}$ are a set of constants specific to H&E coloring for each RGB channel.^27^

### 2.5 Validating histology

After all LSFM examinations were completed, selected tissue samples were further processed for histological analysis and therefore placed in xylene for 1.5 h, embedded in paraffin and cut into 2 µm sections. For further staining, tissue slices which corresponded to the LSFM data were selected out of the sequentially sliced tissue samples. The slices were deparaffinized (60°C, 30 min) and rehydrated by a descending ethanol series. The H&E stain was performed according to the manufacturer's protocol. Immunohistochemistry targeting most of the hematopoetic cells transmembrane protein CD45 was stained in order to detect immune cells within the tissue samples. The rehydrated tissue slices were stained as follows: target retrieval solution (pH 9, 100°C, 20 min), H_2_O_2_ (10 min, room temperature), Seablock (20 min, room temperature), αCD45 (rabbit anti‐human, clone ERP20033, [Abcam, Cambridge, UK] 1:2000, 4°C, overnight), secondary antibody (anti‐rabbit horseradish peroxidase [Histofine, Nichirei Biosciences, Japan]; 30 min, room temperature), finally AEC (3‐amino‐9‐ethylcarbazole) substrate (20 min, room temperature). Washing steps in between were carried out with tris‐(2‐amino‐2‐(hydroxymethyl)‐1,3‐propandiol) buffer (2 × 5 min, room temperature). The tissue slices were covered with corresponding mounting medium and cover glass for evaluation by light microscopy (Zeiss Axioscope II, Carl Zeiss Microscopy GmbH, Germany).

## 3 RESULTS

### 3.1 Prior paraffinization of testicular tissue does not alter autofluorescence properties

As the authors were able to show in a previous study using LSFM, murine testicular tissue, directly fixed and cleared after dissection, has autofluorescence properties enabling morphological identification of the deferent duct, the epididymis, the germinal epithelium with Leydig cell cluster and others.^23^ To evaluate the effect of long‐term preservation of paraffin‐embedded mouse testes on autofluorescence characteristics, a combination protocol involving deparaffinization and subsequent clearing with BABB was implemented.

**FIGURE 1:**
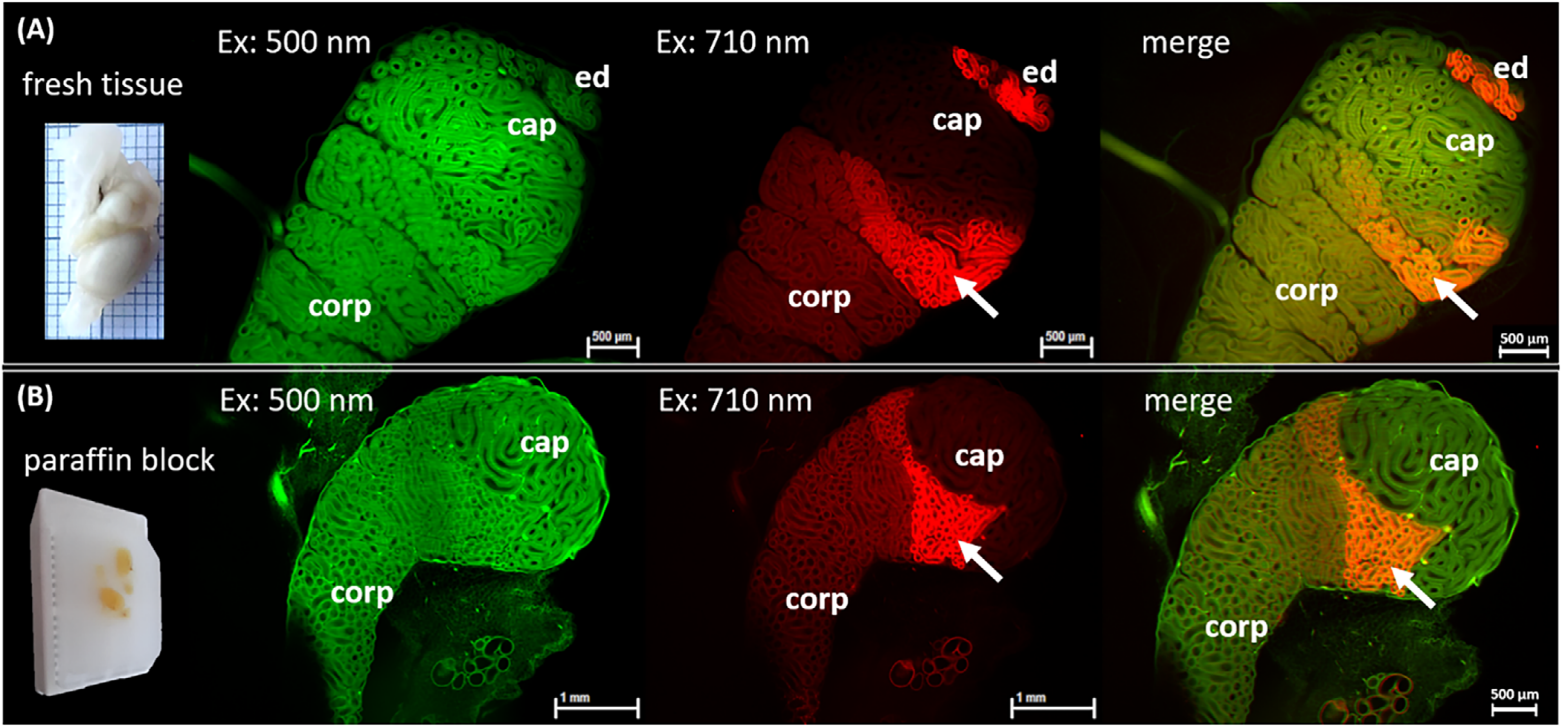
Representative LSFM datasets of fresh (A) and paraffinized (B) murine epididymal ducts. (A, B) 2D views of testes from C57BL/6 mice (four months) show a comparable, homogeneous pattern of autofluorescence signals in the caput (cap) and corpus (corp with the filter set 2 (green). When using the filter combination 5 (red), a pronounced area with strong autofluorescence in the area of the caput epididymidis is recognizable (arrow). There are no significant differences between freshly prepared tissue (A) for LSFM analysis and those from paraffinized testes (B). LSFM, Light sheet fluorescence microscopy.

Samples prepared using this protocol underwent whole scanning using LSFM, with resulting datasets being analyzed and compared with data from our prior study.^23^ In the earlier study, fresh murine testicular tissue was fixed using 37% modified Davidson's fixative, cleared, and stored in BABB pending LSFM scanning. Both sample groups showed adequate transparency within 12 days post‐deparaffinization and were subsequently imaged using LSFM. Various filter set combinations were utilized with the white light laser (Table 1) to excite and detect autofluorescence across the entire testicular volume and a wide wavelength spectrum. The murine testes based on fresh tissue (Figure 1A) demonstrated typical autofluorescence phenomena in the junction of efferent ducts with the epididymis as well as in segments of the caput, which could also be reproduced in the dataset of the deparaffinized testis sample (Figure 1B).

The comparison of the LSFM dataset of the sample prepared from fresh tissue with the dataset of the initially deparaffinized sample showed no differences in the distribution pattern, intensity, and wavelength characteristics of the autofluorescence. This prerequisite ensures that further fluorescence analysis of the human testicular tumor samples could be performed using paraffinized tissue blocks.

### 3.2 Characteristic features of testicular germ cell tumor, assessable in virtual 2D/3D light sheet fluorescence microscopy datasets, are comparable to standard histology

To evaluate the diagnostic value of LSFM data from the punch biopsies of three human GCT specimens, the virtual sections of the acquired 3D datasets were first examined for features of pathological alterations associated with seminomas and mixed GCT.

**FIGURE 2:**
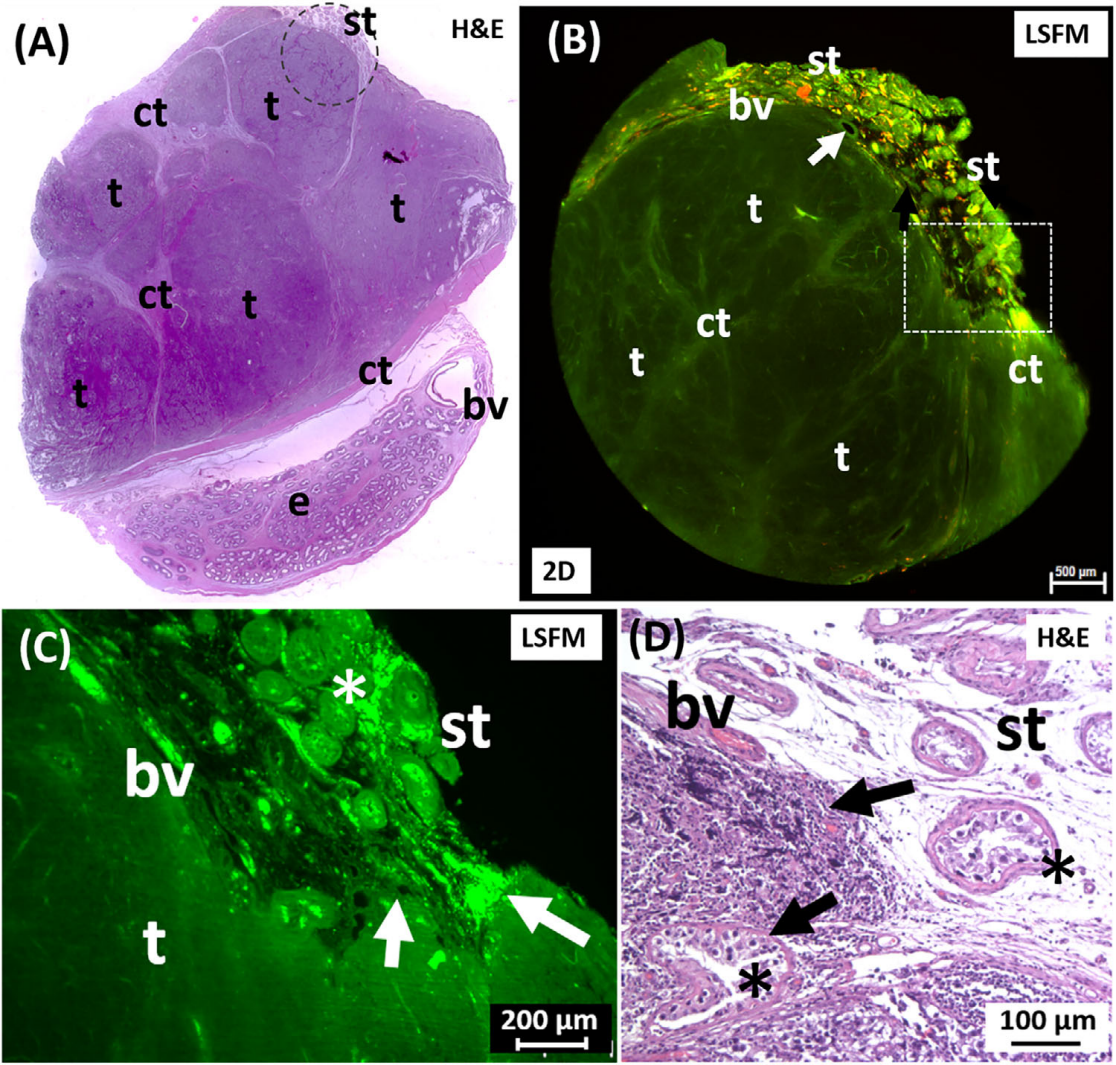
LSFM dataset of an unlabeled seminoma tissue punch compared to H&E appearance. (A) H&E tumor (t) section with connective tissue septa (ct), parts of the epididymis (e) and seminiferous tubules (st). The punch (dashed circle) contains solid tumor tissue (t) and germinal epithelium. (B) LSFM dataset using the filter set 2 (green) and 5 (red) and shows tumor mass (t), connective tissue (ct) and interspersed blood vessels (white arrow). (C) Infiltration of the tumor cells (arrows) into the loose gonadal stroma, note the altered seminiferous tubules (star) and erythrocytes filled blood vessels (bv). (D) The corresponding H&E section confirms the presence of seminoma cells (arrows), surrounding seminiferous tubules, probably with GCNIS morphology (stars). LSFM, Light sheet fluorescence microscopy; H&E, Hematoxylin/Eosin; GCNIS, germ cell neoplasia in situ.

After assessing the corresponding H&E section, the paraffin punch biopsies from tissue blocks of orchiectomy specimens were prepared without any labeling and scanned with LSFM. The images generated by LSFM showed typical morphological features of GCT. In the dataset of the punch biopsy of a seminoma (Figure 2) of a 31‐year‐old patient, adjacent seminiferous tubules (Figure 2A,B) were visible in addition to a solidly grown tumor mass. The seminoma was organized in tumor cell lobules separated by fine fibrotic septa and surrounded by a fine connective tissue capsule. Although the tumor mass and seminiferous tubules appeared separately, there were also infiltrative tumor parts that grew into the area of the tubules (Figure 2C). These tubules were surrounded by loose connective tissue with Leydig cell clusters that showed specific autofluorescence after laser excitation in the near‐infrared wavelength range (ex: 710/75 nm; em: 810/90 nm).

Since these autofluorescence signals could also be detected in the adjacent tumor mass, the infiltrative, displacing growth of the seminoma into the area of the germinal epithelium could be traced and confirmed (Figure 2C). The comparative conventional histological assessment of the tumor validated the findings made by the use of LSFM data (Figure 2D).

**FIGURE 3:**
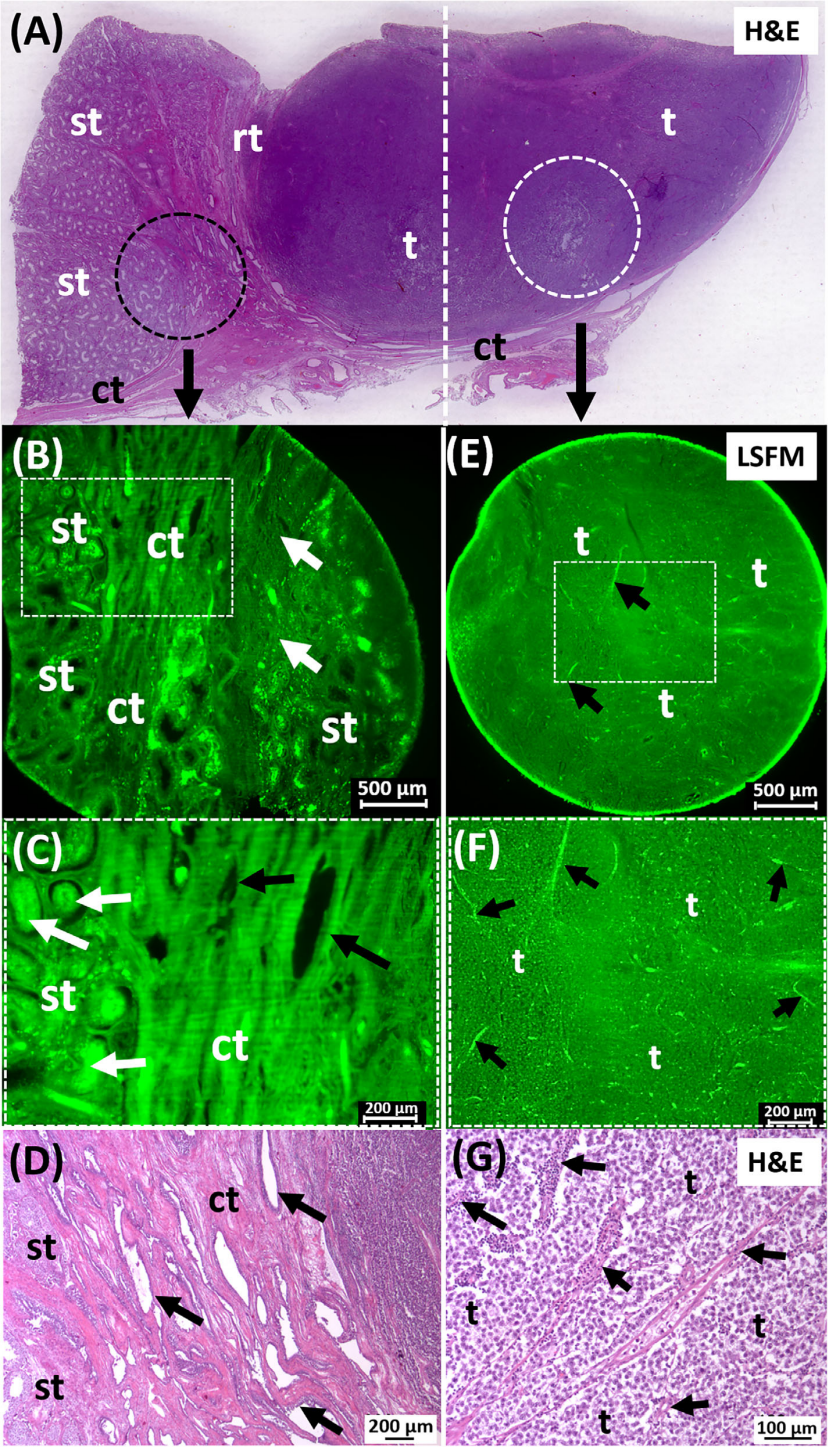
LSFM images of an infiltrating seminoma. (A) The H&E slide shows the tumor mass (t), parts of the rete testis (rt), seminiferous tubules (st), and surrounding connective tissue (ct). (B) An optical LSFM section of a punch (black circle in A) shows the rete testis with stromal cords (ct), testicular tubules and a loose cell mass (arrows). (C) Magnification shows a pagetoid spread of tumor cell clusters (black arrows) into the stroma. Strong fluorescent signals emanate from morphologically altered testicular tubules (white arrows). (D) The histologic preparation correlates with the findings of the pagetoid tumor spread. (E) LSFM data from the second punch (white circle in A) shows a homogeneous distribution of tumor cells (t). (F) Granular cells, fine stromal septa (black arrows) and blood capillaries are clearly visible after magnification. (G) The corresponding H&E section distinguishes between tumor cells (t) and perivascular lymphocytes/plasma cells (arrows). The filter combination 2 was used for LSFM. LSFM, Light sheet fluorescence microscopy; H&E, Hematoxylin/Eosin.

Two tumor punch biopsies from the orchiectomy specimen of a seminoma from a 33‐year‐old patient (Figure 3A–G) were prepared as described, without further labeling, and assessed in more detail by LSFM examination. The LSFM dataset of the first punch biopsy from the area of the rete testis showed a typical intraepithelial, pagetoid spread of the seminoma cells (Figure 3B–D). Here, cell‐rich areas of the tumor alternated with connective tissue strands of the rete testis. Seminiferous tubules interspersed in the stroma and showed different fluorescence properties depending on their morphology. While tubules with a visible lumen were detected with moderate autofluorescence in the low‐wavelength range, pathologically altered tubules without a lumen, were conspicuous by particularly strong fluorescence (Figure 3C). This tumor morphology was also confirmed by a planar histology of the corresponding H&E tumor section (Figure 3D). The second punch biopsy was taken centrally from the solid tumor mass, which was surrounded by a fine connective tissue capsule. The tumor itself was lobularly grown in fine stromal septa (Figure 3E–G). Evenly distributed, small caliber blood vessels were also visualized. Utilizing a higher magnification with 4× objective, a solid, homogeneous tumor growth could be detected, whereby small‐grained tumor cells were individually distinguishable (Figure 3F). Furthermore, a comparative histological examination of the HE section of this tumor revealed not only the presence of seminoma cells but also a large number of small cell lymphocytes infiltrating the seminoma, indicating an inflammatory process (Figure 3G). Both cell types were indistinguishable in the LSFM dataset of the unlabeled biopsy due to the similar nuclear/cytoplasmic ratio (Figure 3F).

**FIGURE 4:**
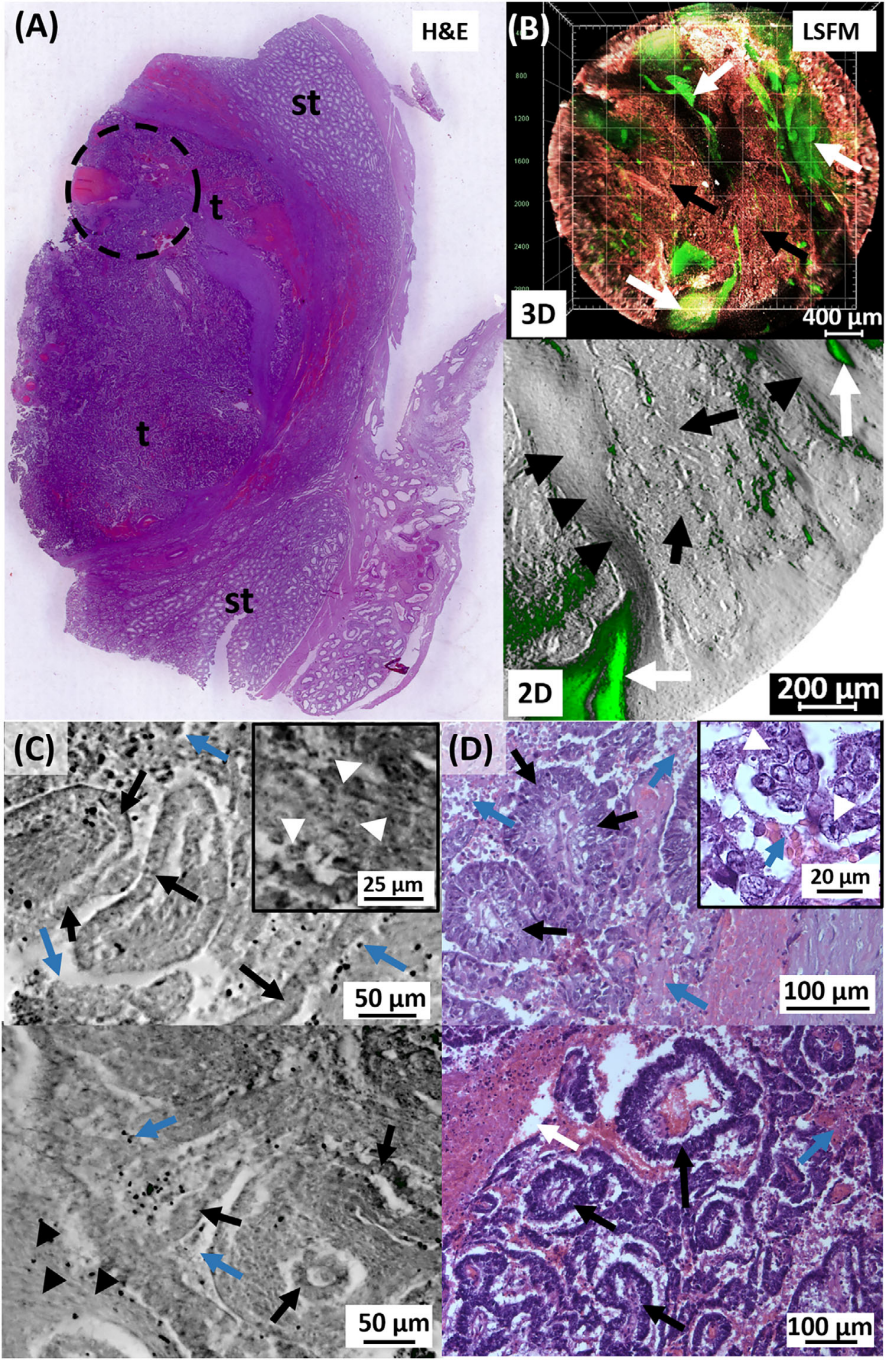
LSFM analysis of mixed testicular tumor. (A) The H&E overview shows the heterogeneous tumor mass (t), and seminiferous tubules (st). (B) The 3D view of the punch biopsy (dashed circle in A) visualizes large blood vessels (white arrows), columnar tumor cell formations (black arrows) and stromal trabeculae (arrowheads). Used filter sets: 2 (green) and 5 (gray inverted). (C) Magnified view of punch biopsy: Top: teratoma portion with tubules of prismatic epithelium, smaller cysts (black arrows) and edematous stroma (blue arrows). Frame: tumor cells with vacuolized cytoplasm, nuclei and nucleoli (arrowheads). Bottom: yolk sac tumor portion with necrotic areas, hemorrhages (blue arrows), and tumor epithelium (black arrows), partly with papillary invaginations. (D) Follow‐up H&E sections confirm epithelial tubules with vacuolized cells (black arrows, arrowheads in frame) in papillary and glandular formations. LSFM, Light sheet fluorescence microscopy; H&E, Hematoxylin/Eosin.

Several punch biopsies were available from a 27‐year‐old patient. The detailed histopathological examination after the orchiectomy led to the diagnosis of a mixed GCT with portions of embryonal carcinoma, teratoma, yolk sac tumor, and chorion carcinoma. From all the material examined, a tumor tissue block was available for LSFM analysis, which contained parts of the teratoma and the yolk sac tumor. The LSFM scans initially showed a pronounced heterogeneity of the punch material (Figure 4).

In addition to large caliber blood vessels that permeated the entire punch, strong stromal strands, peripheral nerves and particularly cell‐rich tumor areas were also noticeable by their autofluorescence properties alone (Figure 4). In particular, the characteristic epithelial morphology of the solidly grown tumor with pleomorphic tumor cells in columns and glandular formations led to the differential diagnosis of an embryonal carcinoma. Since different filter sets were used for the analysis of the tumor punch biopsies in the LSFM, the erythrocytes fluorescing in the short‐wavelength range could be clearly distinguished from the tumor cells fluorescing in the longer‐wavelength range (Figure 4C). In this way, the highly prismatic tumor cells, which exhibited a glandular to cystic growth pattern, could be differentiated down to subcellular resolution. Large cells with vacuolized cytoplasm and clearly euchromatic nuclei with nucleoli could be identified as tumor cells, which corresponded to the teratoma part of this mixed tumor (figure 4C). Rosette‐like, partially cystic tumor cell formations with large, pleomorphic nuclei were also found in the same tumor punch. The presence of these rosettes and papillary invaginations of the tumor cells as well as hemorrhages and necrotic parts corresponded to the proportion of the yolk sac tumor (Figure 4C). These tumor formations were interrupted by connective tissue tracts in the area of the rete testis and could be traced to the altered testicular tubules. Using the LSFM approach, connective tissue of the rete testis, blood vessels and the germ cell epithelium in the seminiferous tubules as well as the solid tumor mass could be detected in all tumor punch biopsies. By comparison with the H&E staining previously performed on the paraffin section, the morphological structures of the tumor punches determined in the LSFM could be clearly validated. (Figure 4D).

### 3.3 The additional cell nucleus staining enables the simulation of conventional H&E stainings in the tomographic LSFM datasets

Although the morphological structures of the previously shown testicular tumors could be visualized in LSFM datasets due to the intrinsic tissue fluorescence without additional staining, the gain of information based on additional nuclear staining in LSFM datasets was evaluated.

For this purpose, a punch biopsy was taken from the paraffin embedded orchiectomy tissue of a 45‐year‐old patient in the area of seminiferous tubules. During sample hydration, this punch was stained in its entirety with the fluorescent dye TO‐PRO‐3 and was further prepared like the unlabeled samples.

The TO‐PRO‐3 dye penetrated the whole punch and stained any cell nuclei, allowing an accurate assessment of cell density and nuclear morphology. The fluorescence maximum of TO‐PRO‐3 is specified in the higher wavelength range (ex: 642 nm; em: 661 nm) and could therefore be discriminated against the intrinsic fluorophores with fluorescence spectra in the low‐wavelength range.

**FIGURE 5:**
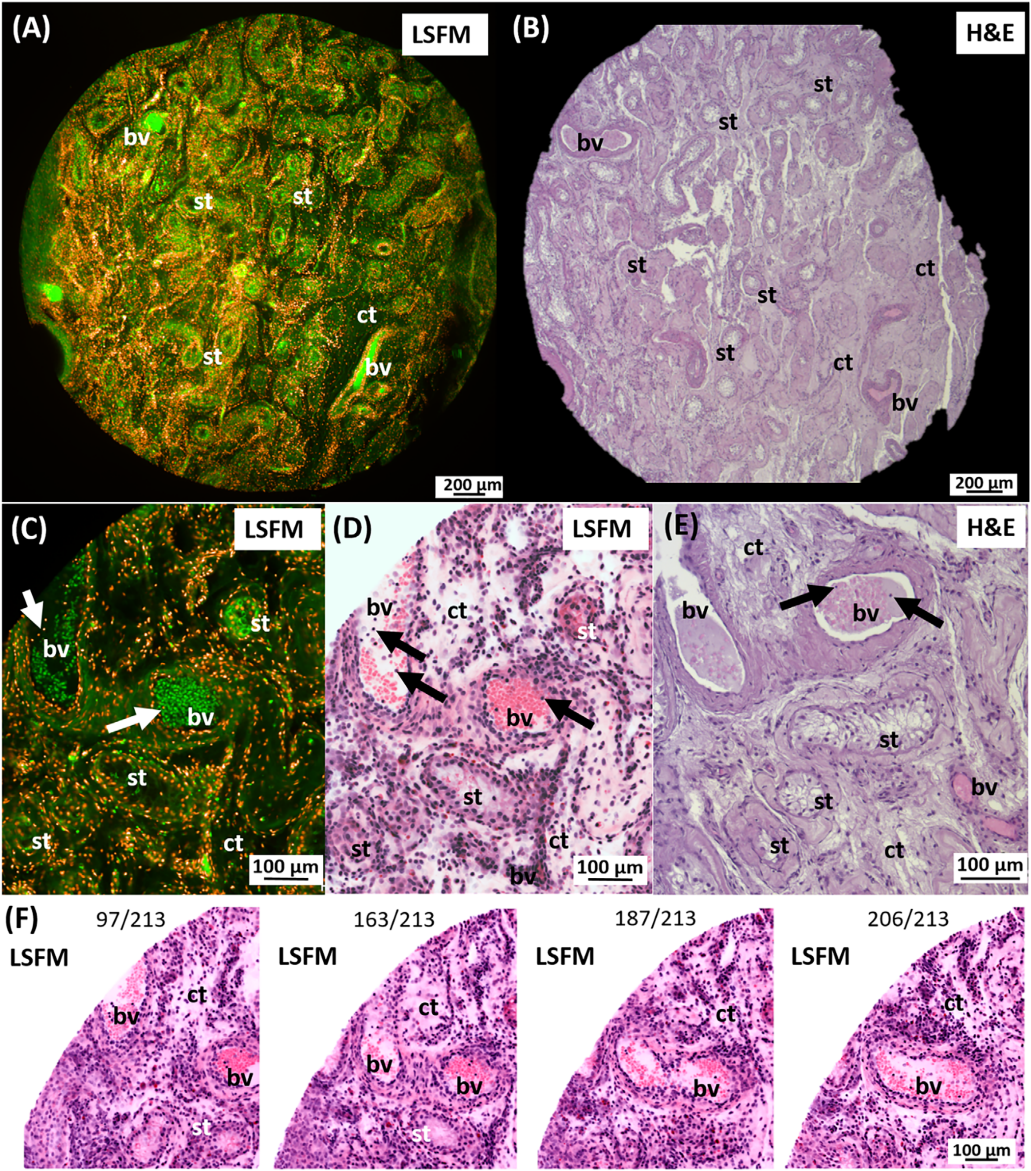
LSFM dataset of a tissue punch stained with TO‐PRO‐3 nuclear staining, transformed into a virtual 2D H&E image. (A) The LSFM 2D slice shows altered seminiferous tubules (st), connective tissue (ct), and interspersed blood vessels (bv). (B) Corresponding H&E section. (C). This LSFM dataset with TO‐PRO‐3 stained cell nuclei (orange) and with highly autofluorescent erythrocytes (arrows) was transformed into false colors mimicking H&E stain and shows nucleated cells (arrows) in addition to the red erythrocytes (D). (E) Corresponding H&E section. (F) False color H&E sections of the tomographic dataset. LSFM images were generated using filter sets: 2 (green) and 4 (orange, TO‐PRO‐3). LSFM, Light sheet fluorescence microscopy; H&E, Hematoxylin/Eosin.

The subsequent LSFM scans were performed with the laser filter set: ex: 630/30 nm; em: 680/30 nm (red) in addition to the usual filter sets in order to capture the fluorescence maximum of the TO‐PRO‐3 dye. With the help of nuclear staining, cell nuclei could be clearly detected in the high‐resolution dataset of a punch biopsy with seminiferous tubules (Figure 5). In combination with the autofluorescence of the basement membranes, the extracellular, collagen‐containing matrix and the nucleus‐free erythrocytes, it was possible to identify the different cell types: Fibrocyte cell nuclei, endothelial cell nuclei and nuclei of the germinal epithelium (Figure 5A,C). This allowed a clear visualization of cell density and pathological morphology within the tubules and surrounding connective tissue, which could be assessed in all tomographic slices of the dataset.

In a second step, the LSFM dataset was virtually processed to match the color spectrum of a typical H&E staining in light microscopy (Figure 5D,F). The virtual dataset showed a larger number of cell nuclei in the tissue than the corresponding H&E section prepared subsequently (Figure 5D,E). This can be explained by the fact that the slice thickness of the LSFM dataset in the *z*‐direction was 4 µm, whereas the slice thickness of a conventional H&E section is 2 µm. Within the dataset of all 213 optical sections generated from the 1 mm thick punch, the presence of nucleated cells in the blood vessels could be tracked over the entire volume with the serial, virtual H&E slices (Figure 5F). The nucleated cells in the tissue punch were identified as immune cells based on their distribution and quantity (Figure 5F). The additional virtual processing of the LSFM dataset thus enabled the histopathological assessment of the tumor tissue punch simulating an H&E staining, but extended by the tomographic information of serial sections of the entire sample volume.

### 3.4 Tomographic dataset of an unlabeled Leydig cell tumor provides more structural and functional information than conventional 2D pathohistology alone

**FIGURE 6:**
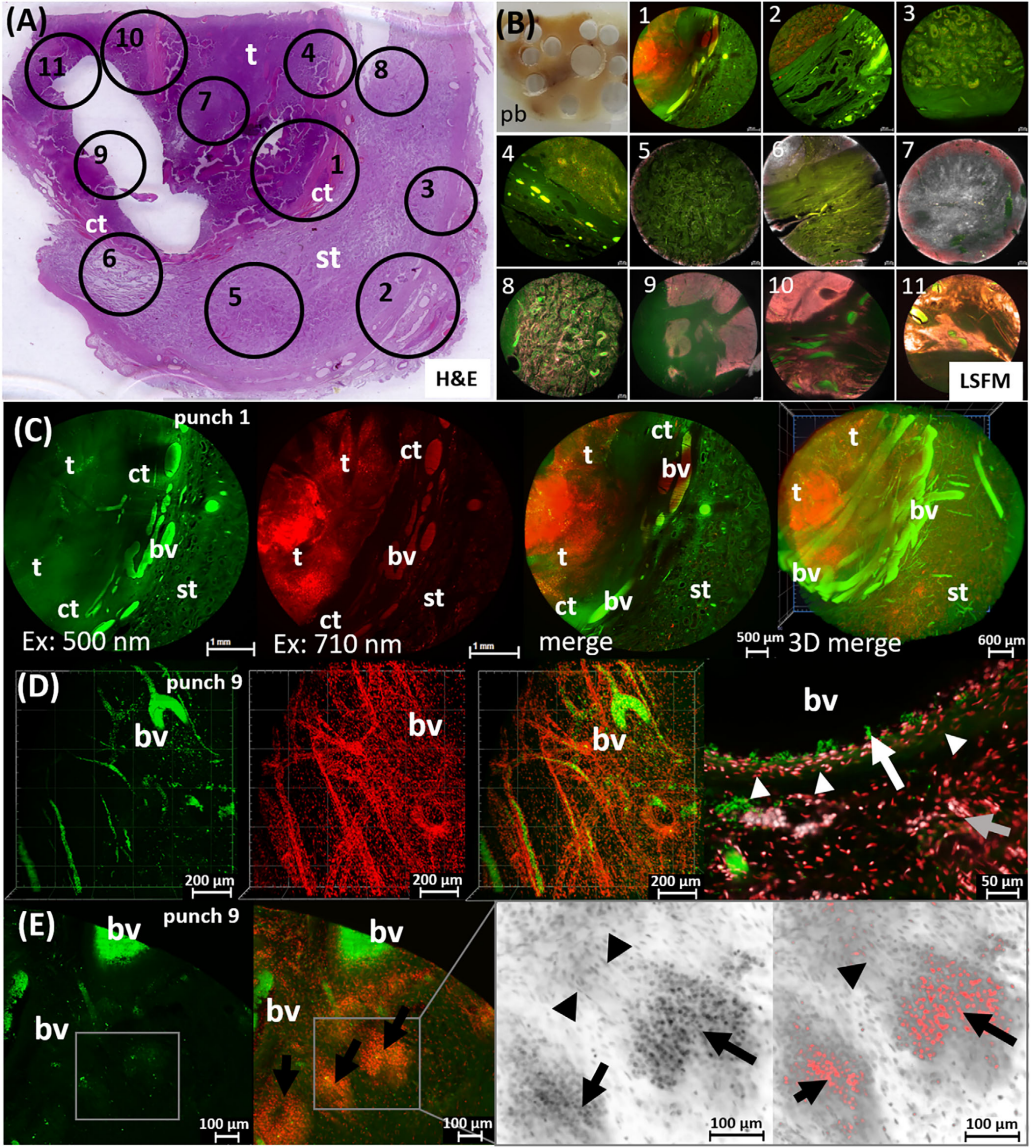
Comprehensive LSFM analysis of a Leydig cell tumor (LCT) by scanning tissue punches from one paraffin block (pb). (A, B) The H&E section with tumor mass (t) connective tissue strands (ct) and seminiferous tubules (st). (B) Punches 1–5: unlabeled, punches 6–11: stained with TO‐PRO‐3. (C) LSFM data of punch 1 show heterogeneously distributed long wavelength fluorescence signals, indicating the presence of Leydig cells. Blood vessels (bv), connective tissue and seminiferous tubules appear in the low‐wavelength range. Filter set 2 (green) and 5 (red). (D) TO‐PRO‐3 stains cell nuclei in endothelial strands close to erythrocytes (green). At highest magnification, smooth muscle cells of the vessel wall (arrowheads), erythrocytes (arrow), subjacent fibrocyte nuclei and cloudy tumor cell accumulations (grey arrow) can be assessed. (E) Nuclear staining reveals tumor cell nests in the surrounding stroma. The magnification (frame) shows cell nuclei of fibrocytes (arrowheads) and tumor cells (arrows) with cloudy surroundings. Used filter sets: 1 (green); 4 (orange) and 5 (inverted grey). LSFM, Light sheet fluorescence microscopy; H&E, Hematoxylin/Eosin.

Unlike the seminomas and embryonal tumors presented in the previous chapter, the benign LCT presented here is a gonadal stromal tumor. The tumor sample came from an orchiectomy in a 45‐year‐old patient. The H&E overview of the entire tumor section showed not only the tumor mass but also abundant pathologically altered germinal epithelium, both tissue portions were separated from each other by highly vascularized stromal strands (Figure 6A).

In order to analyze the autofluorescence properties of this type of rare testicular stromal tumor as comprehensively as possible, 11 tissue punches were taken from a paraffin‐embedded LCT (Figure 6B) and processed for LSFM analysis. Five tissue punches from the area of the solid tumor mass but also from the area of the seminiferous tubules were prepared unlabeled to detect pure autofluorescence, punches 6–11 were additionally treated with TO‐PRO‐3 nuclear stain and all were cleared and scanned by LSFM. The first tissue punch, which was unlabeled, contained parts of the solid tumor mass as well as the area of the seminiferous tubules (Figure 6C). Both parts could be well discriminated by LSFM scan even in the low‐wavelength range through the blood vessels with the strongly autofluorescent erythrocytes within the connective tissue. In particular, the 3D image of the entire punch showed the spatial orientation of the blood vessels within the tissue—from the large‐caliber vessels of the connective tissue portion to the delicate, small‐caliber vessels that supplied the seminiferous tubules to the vascular networks of the tumor mass. In the higher wavelength range, however, cloudy autofluorescence signals could be detected within the tumor mass, which differed in distribution and intensity (Figure 6C). This intrinsic fluorescence was also noticeable as dot‐shaped nests between the seminiferous tubules and could most likely be addressed as Leydig cell clusters due to their location.

The blood vessels of different calibers were also analyzed in LSFM datasets of the punches that had been stained with TO‐PRO‐3 (Figure 6D). The staining enabeled to visualize the endothelial cell nuclei surrounding the vessels, which could be clearly identified by adding the information of the autofluorescent erythrocytes. Moreover, the absence of the typical erythrocyte fluorescence allowed an unambiguous determination of fibrocyte nuclei. This resulted in a precise representation of the fine, dense vascular network that is typical of tumors with the endocrine activity. At the highest possible magnification (Figure 6D), in addition to erythrocytes and endothelial cell nuclei, the nuclei of the smooth muscle cells of the vessel wall were also detected, as well as evenly distributed fibrocytes of the underlying connective tissue. In this stromal tissue, in addition to blood vessel capillaries, strongly fluorescent cell nucleus clusters were localized, which could be addressed as tumor cell clusters due to their morphology.

These tumor cell accumulations could be observed in several TO‐PRO‐3 stained punches (Figure 6B, 9–11) as distinct, highly fluorescent nuclei clusters of different sizes. An LSFM analysis of the TO‐PRO‐3‐stained punch 9 revealed the image of distinctly stained cell nuclei of tumor cell clusters interspersed in the connective tissue with homogeneously distributed fibrocyte nuclei (Figure 6E), with the filter combination of ex: 630/30 nm; em: 680/30 nm.

At the same time, a less distinct, cloudy fluorescence pattern around the tumor cell nuclei could be detected with the filter combination ex: 710/75 nm; ex: 810/90 nm, which indicates the presence of Leydig cells with its typical intrinsic fluorescence.

**FIGURE 7:**
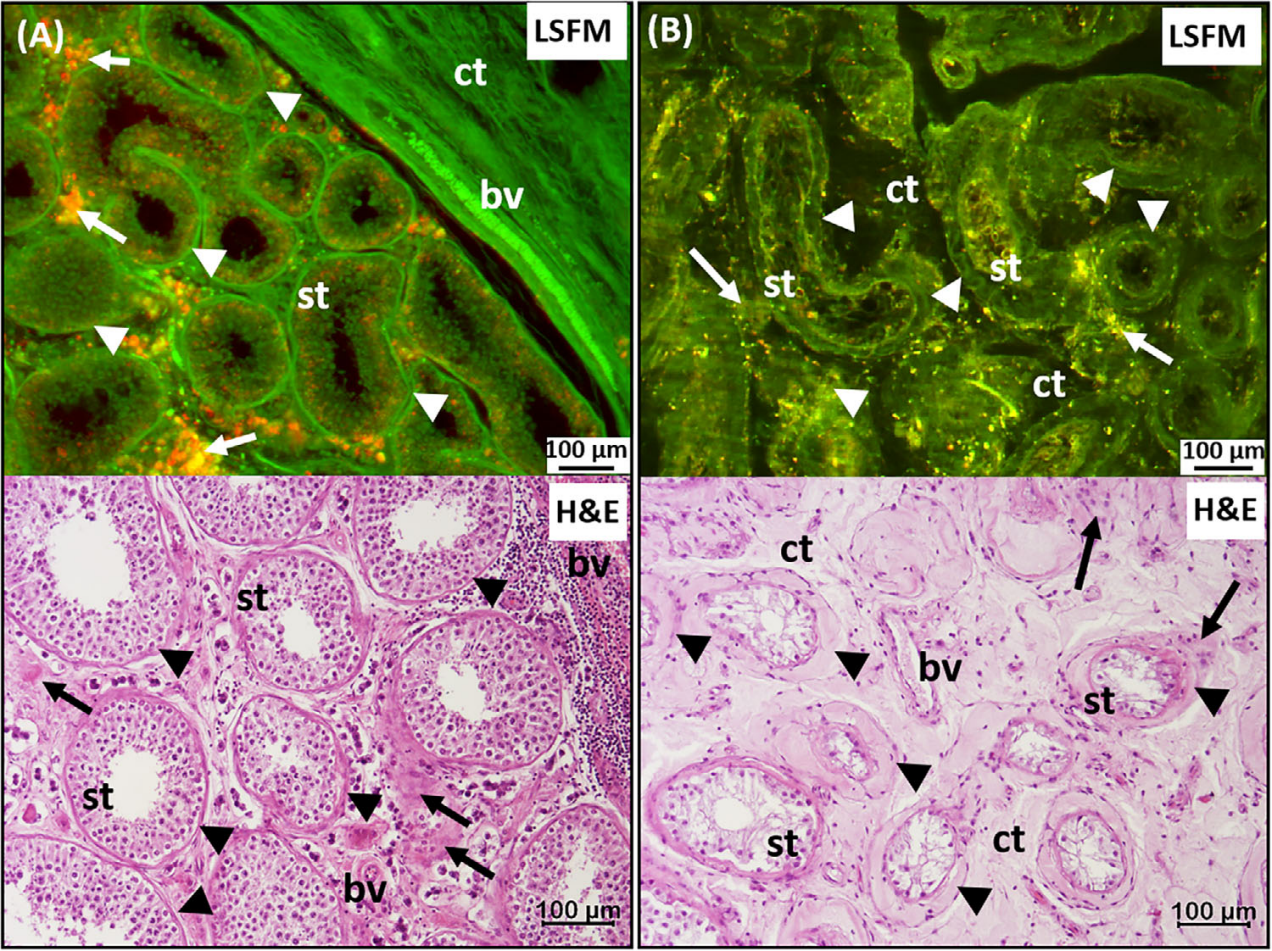
Unlabeled LSFM images provide additive information about Leydig cell appearance than conventional histology alone. (A) LSFM dataset of unaltered seminiferous tubules (st) with regular lumina, germinal cells surrounded by a basement membrane (arrowheads) and connective tissue (ct) and blood vessels (bv). Autofluorescent Leydig cells (orange, arrows) are located in the stroma. (A, bottom) Corresponding H&E section. (B) In comparison, an LSFM image of a LCT with features of azoospermia is shown: “empty” seminiferous tubules with thickened basement membranes (arrowheads) and loose connective tissue. Weak fluorescent Leydig cells (arrows) are rarely interspersed in the stroma. Although the Leydig cells (arrows) are visible in the corresponding H&E section (B, bottom), no information is given about their function. LSFM images were composed of filter sets: 2 (green) and 5 (red). LSFM, Light sheet fluorescence microscopy; H&E, Hematoxylin/Eosin.

In the unlabeled punch 3 of the same tumor, which was taken exclusively from the area of the seminiferous tubules (Figure 7), morphological alterations of the germinal epithelium could be detected after LSFM analysis, which supported the finding of azoospermia (Figure 7B).

The small seminiferous tubules, located in the loose connective tissue were characterized by a thickened basement membrane, which was clearly visible in the low‐wavelength range due to the collagen content (Figure 7B). Furthermore, only individual cells of the germinal epithelium were visible, which showed a vacuolized, “empty” cytoplasm. These pathological characteristics of azoospermia were also visible in the H&E staining, previously prepared of the intact paraffin block. In the planar histology, Leydig cell clusters were also detected in the peritubular connective tissue, which were not visible in the corresponding LSFM dataset. The typical intrinsic fluorescence of the Leydig cells in the longer wavelength range was either absent or was only focally present in small spots.

The LSFM data suggest that the lack of detection of Leydig cell clusters is not due to a lack of Leydig cells, but rather to a lack of typical synthesized compounds of these cells. The LSFM data of a morphologically and functionally inconspicuous germinal epithelium (Figure 7A) showed densely packed, cell‐rich seminiferous tubules with highly fluorescent, regular basement membranes. Individual cell nuclei were also detectable within the germinal epithelium, although no further tissue staining was performed. Abundant blood capillaries supplied the peritubular connective tissue and the nearby Leydig cell clusters could be anticipated by the presence of strong intrinsic fluorescence in the longer wavelength range. The morphological structures were also found in the H&E staining of a planar tumor section of the same region. The LSFM data thus extended the histological picture of the germinal epithelium with information on the likely presence of functional compounds of the endocrine Leydig cells.

Subsequent histological and immunohistochemical (IHC) examination of the tissue punches is feasible after sample preparation for LSFM.

**FIGURE 8:**
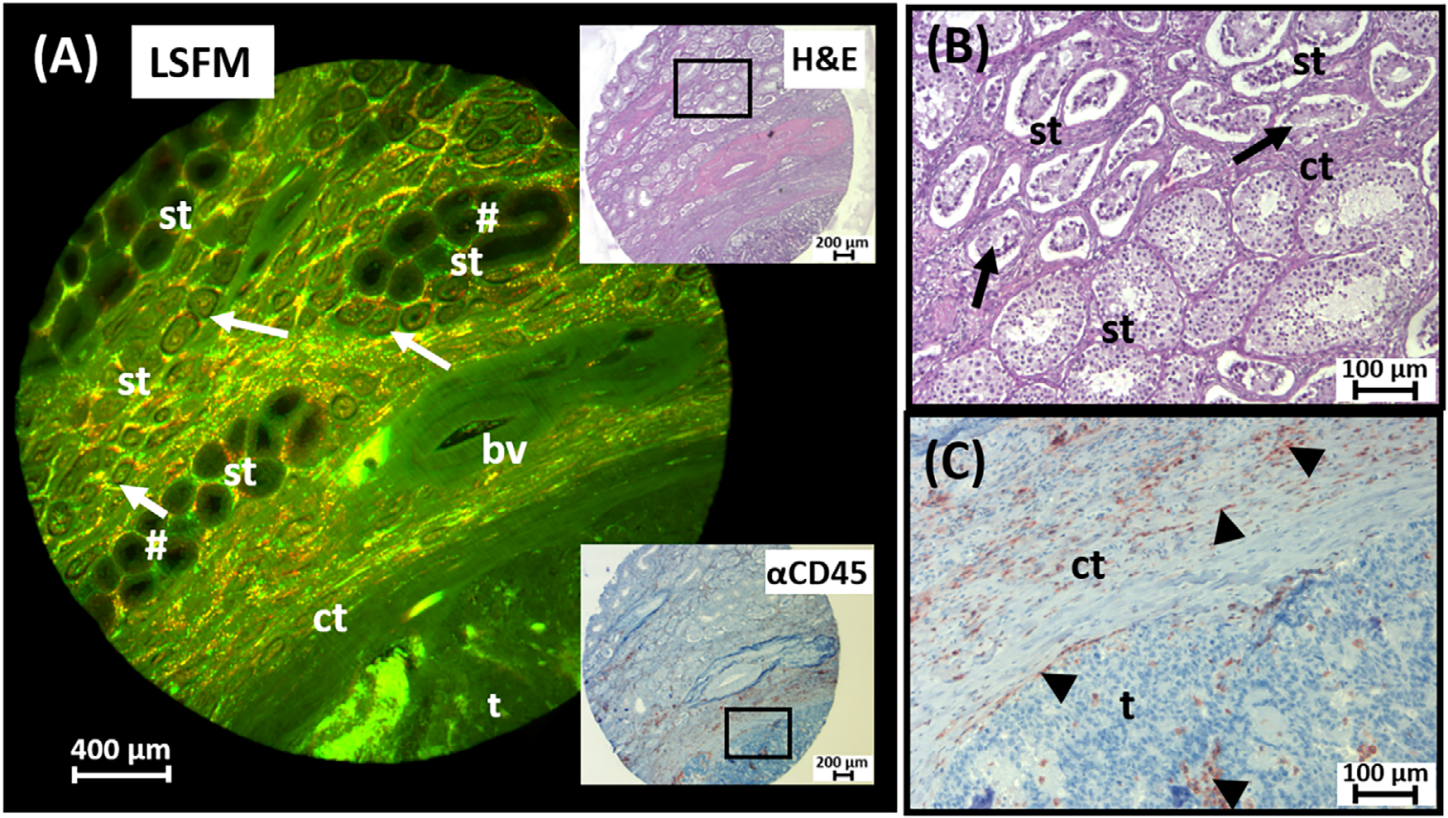
Successful histological and immunohistochemical (IHC) staining of previously LSFM‐scanned tissue punches. (A) Tomographic section of an LSFM dataset of a seminoma punch containing pathologically altered seminiferous tubules (st) with narrowed lumen as germ cell neoplasia in situ (arrows) as well as wide lumina of unaltered seminiferous tubules (#), connective tissue tracts (ct) and blood vessels (bv). The image was created with the filter set 2 (green) and 5 (red). (B) Conventional H&E staining of the corresponding section of the re‐paraffinized tissue punch is comparable to (A). (C) A consecutive IHC approach after LSFM analysis with an antibody against CD45 shows the specific binding to immune cells (arrowheads). LSFM, Light sheet fluorescence microscopy; H&E, Hematoxylin/Eosin.

In order to test whether the evaluation of the biopsy punches by LSFM can be followed by a new histopathological analysis, the described workflow was extended by re‐embedding the cleared and scanned tissue sample in paraffin again. The tissue punch of an embryonal carcinoma, which had already been prepared, cleared, and scanned using LSFM, was paraffinized again after LSFM analysis, cut and the tissue sections were chemically and immunohistochemically stained (Figure 8). The LSFM dataset (Figure 8A) showed not only proportions of inconspicuous seminiferous tubules but also structures that indicated a morphological change in the germinal epithelium, which was indicative of impaired spermatogenesis. In addition to various luminal diameters of the tubules and intervening stromal strands, parts of the solidly growing tumor were also detected in the punch. The H&E staining of the tissue punch after re‐embedding of the cleared tissue showed a corresponding morphological pattern, both in the area of the tumor and in the area of the seminiferous tubules (Figure 8B). This enabled a clear differentiation of different sperm stages, but also of the altered seminiferous tubules in particular and various tissue components such as connective tissue, blood vessels and tumor cells. IHC staining with an antibody against the CD45 protein showed the accumulation of immune cells in the area of the testicular stroma and between the loosely packed tumor cells (Figure 8C), in accordance with its specificity. The results show that the workflow of deparaffinization, clearing and LSFM analysis enables subsequent chemical and IHC staining of the later re‐paraffinized samples.

## 4 DISCUSSION

The present data show a 2D/3D virtual histology approach for the detailed visualization of relevant testicular tumor features using LSFM on paraffin embedded testicular tumor biopsies.

Using LSFM as a tomographic microscopy technique, cleared tissue biopsies can be imaged at the highest possible resolution with a voxel size of 0.217 µm in the *X*/*Y*‐direction, and 1–2 µm in the *Z*‐direction. In this study, 2D tomographical images as well as 3D volumes revealed the exact distribution of blood vessels, connective tissue strands of the rete testis, the seminiferous tubules with the germinal epithelium and the tumor mass itself. This provides a comprehensive picture of the pathological alterations in these individual structures and tissue components.

A straightforward method of deparaffinizing tissue paraffin blocks^26^ as well as the clearing protocol was adapted to the testicular tumors. 3D or sequential tomographic datasets could be assessed in all arbitrary angles without labeling. According to van Ineveld et al., the use of previously formalin fixed paraffin‐embedded (FFPE) tissue blocks for retrospective 3D analysis offers several advantages: (I) numerous, excellently documented FFPE tissue samples are available due to conventional pathologic findings, which can be used for extensive studies. (II) Compared to a few planar sections, the tissue volumes used provide many times more information. (III) The knowledge of diagnosis and individual outcome provides new tools for identifying meaningful imaging tumormarkers.^28^ Because complete clearing is a prerequisite for LSFM and the generation of high‐quality datasets, there is much research into establishing clearing protocols, but these need to be adapted to individual tissue types, consistency, and composition. An effective reduction of light scattering was achieved in particularly collagen‐rich, desmoplastic tissues but also cell‐rich structures, such as those of the germinal epithelium, by methods of solvent‐based clearing.^22^, ^23^, ^29^ The use of BABB led to tissue transparency of the tumor tissue, which ensured that the entire tumor punch could be visualized in 3D. This organic solvent preserves the tissue at the same time and therefore makes it possible to measure samples in several scanning sessions.^30^

BABB clearing did not interfere with intrinsic fluorophores^31^ and resulted in a clear autofluorescence image in the LSFM dataset. Thus, a clear differentiation of pathologically altered and anatomically unaffected structures of the testicular tumor tissue punches was possible by LSFM. The high resolution of the data allowed a qualitative assessment of the tumor tissue down to the cytoplasmic level, which was confirmed by the subsequent 2D histology. In a previous study in mouse testes, we showed that blood vessels, Leydig cells, epididymis, and other testicular structures can be clearly visualized due to their specific intrinsic fluorescence profile.^23^ In the present study, we were able to show that deparaffinized mouse testes have the same intrinsic fluorescence properties as mouse testes that were cleared and analyzed immediately after dissection.

This was an important prerequisite for the use of paraffin‐embedded testicular tumor tissue in this retrospective approach. The precise differentiation of intrinsic fluorescence allowed the visualization of azoospermia within an LCT tissue punch, characterized by the absence of germ cells, thickening of the tubular basement membrane and reduced presence of Leydig cell clusters within the germinal epithelium.

The different autofluorescence behavior of altered and unaffected tissues thus indicated functional changes that have an influence on the composition of the germinal epithelium. The intrinsic fluorescence, which was distributed very heterogeneously in the tumor mass in a LCT tissue punch, could thus be used to identify mechanisms of clonal selection. As hormonally active tumors, the question remains whether typical clinical symptoms such as gynecomastia or the increase in steroid hormones in the patient's serum^8^, ^32^, ^33^ correlate with the density and distribution of autofluorescent structures in the entire tissue punch. A clear indication that this autofluorescence profile is due to synthesized steroidal hormones is still pending^34^ and is the subject of further investigations. This information has the potential to establish new, 3D imaging tumor markers that can contribute to the understanding of tumor development, but also to diagnostics and prognostics.

The potential of label‐free virtual histology using LSFM has already been demonstrated in a number of studies for 3D imaging of organs and tumors.^21^, ^35^, ^36^ The mesoscopic imaging approach of the human pancreas with its islets of Langerhans and tumorous areas in samples of pancreatic ductal adenocarcinoma by Hahn et al. showed that pathological tissue exhibits characteristic, altered intrinsic fluorescence signals, which can therefore be used to detect the tumor microenvironment and tumor margin.^36^

The intrinsic fluorophores depict collagen‐associated morphological structures such as blood vessels, connective tissue or the basement membranes of the seminiferous tubules. Whether the autofluorescence signals of the basement membranes of the seminiferous tubules are those of collagen IV or collagen I cannot be conclusively determined on the basis of the LSFM signal we detected. The work by Alfano et al. shows that azoospermia leads to increased collagen I deposition in the basement membrane.^37^ Further research is needed to investigate whether it is possible to discriminate between the LSFM autofluorescence signals of different collagens. Helpful approaches can also be found in the work of Timin and Milinkovitch, who use the dye fast green to stain and analyze collagens also by LSFM.^38^

Although the visualization of seminiferous tubules is very accurate, the individual cell nuclei were rather discreet; a step of fluorescence staining of the cell nuclei was implemented during the clearing protocol.

The TO‐PRO‐3 dye and its alternatives stains all cell nuclei non‐specifically and thus enables statements regarding cell density and cell nucleus morphology and is able to map tumor heterogeneity in an entire volume much more accurately.^25^, ^39^ Due to the fluorescence properties of the nuclear dye,^40^ it was possible to discriminate against the intrinsic fluorophores in the low‐wavelength range. The combination of nuclear staining with the tissue specific autofluorescence thus connects the advantages of conventional histology with the tomographic possibilities of virtual serial sections of the entire tissue punch volume. This enables, for example, the detection of nucleated tumor cells in blood vessels with nucleus‐free erythrocytes across all virtual projections of the sample.

With or without nuclear staining, our data show that an assessment of the peritumoral seminiferous tubules and thus of spermatogenesis and azoospermia is possible at the cellular level and can be reproduced in the entire dataset. As a known pivotal parameter of diagnosis, the evidence of a GCNIS which manifests itself in the tubules, indicates the presence of a GCT^41^ and influences all subsequent diagnosis and treatment.^42^, ^43^ Therefore, a tomographic analysis of the whole punch biopsy provides a comprehensive contribution to a detailed diagnosis due to a maximization of information. Similar studies showed the benefits for comprehensive metastasis detection in human lymph nodes.^44^

The assessment of the tumor margins, the precise differentiation of the heterogeneous teratoma from epidermal cysts or the determination of seminoma versus non‐seminoma portions of a testicular tumor are also diagnostic challenges^42^ that can be clarified more comprehensively using tomographic tumor datasets than isolated planar histological slides. After re‐embedding the cleared tissue samples and microtome sectioning, further IHC analysis was successful, which was confirmed by consecutive H&E and anti‐CD45 antibody staining. We observed that both, the binding specificity and the staining pattern did not change compared to standard histology. Performing standard histopathology following clearing plays a decisive role in translation, that is, in the implementation of LSFM in clinical practice as light sheet guided histology.

The new WHO classification of testicular tumors 2022^45^, ^46^ shows that the classification of testicular tumors is subject to constant amendments based on new histomorphological and diagnostic findings. The tomographic and 3D visualization of novel auto/fluorescence parameters using LSFM analysis can make an important contribution to the subtype classification of seminomas and gonadal stromal tumors. A major development in histopathology is the deep learning assisted analysis of patient samples, for example, for prostate cancer.^47^, ^48^ However, reliable structure recognition urgently requires a lot of data and not randomly selected planar tissue sections. Here, 3D microscopy with its tomographic datasets offers an ideal basis and additional benefits for comprehensive data analysis.^49^

In summary, this study shows that virtual 3D and tomographic 2D histopathological analysis is possible with a straightforward clearing protocol of already paraffin embedded tumor tissue blocks of the human testis. The clinical implementation of the presented LSFM method for the assessment of testicular biopsies with altered tissue structures and cellular organizations is thus feasible. Even if LSFM means the implementation of an adapted workflow for sample preparation and data acquisition in the future, the quality and evaluation of the datasets corresponds to the comprehensive diagnosis of sequential serial sections and is therefore not limited to experienced pathologists.

It has the potential to contribute in combination with conventional histology to a comprehensive diagnosis, identification of novel imaging tumor markers and new insights into the development of the pathological changes of the complex male reproductive organ.

## AUTHOR CONTRIBUTIONS

Diana Pinkert‐Leetsch and Jeannine Missbach‐Guentner wrote the manuscript, performed the mouse dissections, designed the study, analyzed and arranged the data. Ahmad Kareem prepared some of the samples and carried out LSFM scans. Simon F. Merz and Marc Teckentrup provided the TO‐PRO‐3 stain, did the image post processing of the data for virtual H&E stain. Felix Bremmer and Alexander Fichtner provided samples, discussed the diagnoses. Jeannine Missbach‐Guentner analyzed the histological data and supervised the work. All authors supported the writing and revised the final version of the manuscript.

## CONFLICT OF INTEREST STATEMENT

The authors declare no conflicts of interest.
